# Coronary fractional flow reserve measurements of a stenosed side branch: a computational study investigating the influence of the bifurcation angle

**DOI:** 10.1186/s12938-016-0211-0

**Published:** 2016-08-05

**Authors:** Claudio Chiastra, Francesco Iannaccone, Maik J. Grundeken, Frank J. H. Gijsen, Patrick Segers, Matthieu De Beule, Patrick W. Serruys, Joanna J. Wykrzykowska, Antonius F. W. van der Steen, Jolanda J. Wentzel

**Affiliations:** 1 Department of Cardiology, Biomedical Engineering, Erasmus MC, Rotterdam, The Netherlands; 2 Laboratory of Biological Structure Mechanics (LaBS), Department of Chemistry, Materials and Chemical Engineering “Giulio Natta”, Politecnico di Milano, Milan, Italy; 3 IbiTech-bioMMeda, Department of Electronics and Information Systems iMinds Medical IT, Ghent University, Ghent, Belgium; 4 The Heart Center, Academic Medical Center, University of Amsterdam, Amsterdam, The Netherlands; 5 FEops bvba, Ghent, Belgium; 6 International Centre for Circulatory Health, NHLI, Imperial College London, London, UK

**Keywords:** Coronary bifurcation, Fractional flow reserve, Pressure drop, Helicity, Computational fluid dynamics, Mathematical model

## Abstract

**Background:**

Coronary hemodynamics and physiology specific for bifurcation lesions was not well understood. To investigate the influence of the bifurcation angle on the intracoronary hemodynamics of side branch (SB) lesions computational fluid dynamics simulations were performed.

**Methods:**

A parametric model representing a left anterior descending—first diagonal coronary bifurcation lesion was created according to the literature. Diameters obeyed fractal branching laws. Proximal and distal main branch (DMB) stenoses were both set at 60 %. We varied the distal bifurcation angles (40°, 55°, and 70°), the flow splits to the DMB and SB (55 %:45 %, 65 %:35 %, and 75 %:25 %), and the SB stenoses (40, 60, and 80 %), resulting in 27 simulations. Fractional flow reserve, defined as the ratio between the mean distal stenosis and mean aortic pressure during maximal hyperemia, was calculated for the DMB and SB (FFR_SB_) for all simulations.

**Results:**

The largest differences in FFR_SB_ comparing the largest and smallest bifurcation angles were 0.02 (in cases with 40 % SB stenosis, irrespective of the assumed flow split) and 0.05 (in cases with 60 % SB stenosis, flow split 55 %:45 %). When the SB stenosis was 80 %, the difference in FFR_SB_ between the largest and smallest bifurcation angle was 0.33 (flow split 55 %:45 %). By describing the ΔP_SB_−Q_SB_ relationship using a quadratic curve for cases with 80 % SB stenosis, we found that the curve was steeper (i.e. higher flow resistance) when bifurcation angle increases (ΔP = 0.451*Q + 0.010*Q^2^ and ΔP = 0.687*Q + 0.017*Q^2^ for 40° and 70° bifurcation angle, respectively). Our analyses revealed complex hemodynamics in all cases with evident counter-rotating helical flow structures. Larger bifurcation angles resulted in more pronounced helical flow structures (i.e. higher helicity intensity), when 60 or 80 % SB stenoses were present. A good correlation (R^2^ = 0.80) between the SB pressure drop and helicity intensity was also found.

**Conclusions:**

Our analyses showed that, in bifurcation lesions with 60 % MB stenosis and 80 % SB stenosis, SB pressure drop is higher for larger bifurcation angles suggesting higher flow resistance (i.e. curves describing the ΔP_SB_−Q_SB_ relationship being steeper). When the SB stenosis is mild (40 %) or moderate (60 %), SB resistance is minimally influenced by the bifurcation angle, with differences not being clinically meaningful. Our findings also highlighted the complex interplay between anatomy, pressure drops, and blood flow helicity in bifurcations.

## Background

Intracoronary hemodynamics can directly be assessed during percutaneous coronary intervention (PCI) using sensor-equipped guide wires, measuring pressure and/or flow [1]. Pressure and/or flow measurements in stenosed arteries have provided us a profound understanding of the coronary physiology [1]. Fractional flow reserve (FFR), defined as the ratio between the mean distal stenosis and mean aortic pressure during maximal hyperemia, has shown to be a valuable tool to assess the functional severity of coronary stenoses in daily clinical practice. Multiple (randomized) trials, including the landmark ‘FAME’ trial, have shown that FFR-guided PCI improves patient outcomes with respect to relief of angina complaints and the necessity of (repeat) angiography [1–4]. Combined use of FFR with coronary flow reserve measurements may provide the clinician an even better understanding of the functional severity of a coronary stenosis and its prognosis [5, 6]. In contrast, FFR guidance for side branch (SB) lesion PCI did not show clinical benefit compared to angiography-guided SB interventions [7, 8]. However, trials on FFR treatment guidance were not specifically designed for bifurcation lesions.

Also after treatment of bifurcation lesions some counter-intuitive FFR measurements have been observed. Treatment of the main branch (MB) in a bifurcation region with a small angle often results in SB compromise, whereas that is less often the case for large bifurcation angles [9]. However, the FFR after treatment was much less compromised for the small angle bifurcations compared to the large bifurcation angles. These contrasting findings imply that coronary hemodynamics and physiology in bifurcations is more complex than in non-bifurcation segments.

Based on the observations described before, we hypothesize that distal bifurcation angle on itself might play a major role in determining SB FFR values. Computational fluid dynamics (CFD) has been demonstrated to be an effective tool to study the hemodynamics of coronary bifurcations, allowing to investigate multiple scenarios characterized by different anatomy and flow conditions [10–17]. Therefore, we performed CFD simulations on a population-based coronary bifurcation model of the left anterior descending (LAD)—first diagonal branch with varying distal bifurcation angles to investigate the influence of the bifurcation angle on the intracoronary hemodynamics, including pressure drops and FFR, of SB lesions.

## Methods

### Coronary bifurcation model

**Fig. 1:**
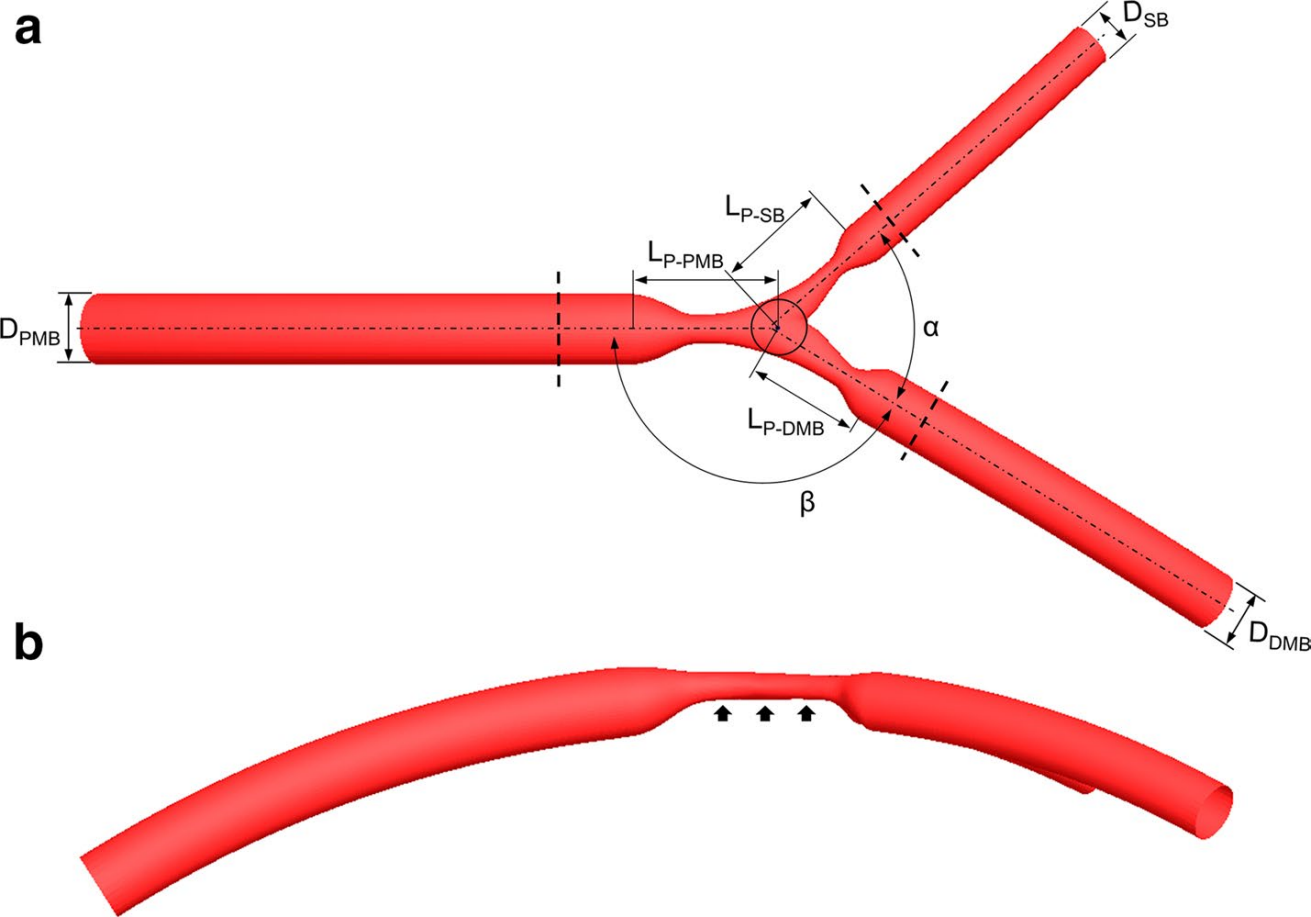
Parametric coronary bifurcation model that represents the left descending coronary artery with its first diagonal branch: *top* (**a**) and *lateral* (**b**) view. *D*_*PMB*_ proximal main branch diameter, *D*_*DMB*_ distal main branch diameter, *D*_*SB*_ side branch diameter, *α* distal angle, *β* main branch angle, *L*_*P−PMB*_ plaque length in the proximal main branch, *L*_*P−DMB*_ plaque length in the distal main branch, *L*_*P−SB*_ plaque length in the side branch. The *black arrows* at the plaque location in (**b**) highlights the plaque eccentricity. *Dashed lines* indicate the locations where pressure were measured for FFR calculations

A parametric coronary bifurcation model that represents the LAD with its first diagonal branch was created using the open-source software PyFormex (http://www.nongnu.org/pyformex/) (Fig. 1a). The model has a proximal main branch (PMB) diameter of 3.30 mm [18]. The diameters of the distal main branch (DMB) and the SB obeyed Finet’s law [19] and they were set as 2.77 mm and 2.10 mm, respectively. Three different distal angles (α; 40°, 55°, and 70°) were chosen according to the studies by Onuma et al. [20] and Godino et al. [21]. The PMB to DMB angle (β) was set to 150° [21]. The PMB segment length (from the inlet cross-section to the stenosis starting point) is equal to eight diameters, i.e. 26.4 mm. This value is in accordance with the measurements by Yamamoto et al. for the human proximal LAD segment, in which a length of 26.6 ± 9.3 mm was measured (n = 101) [22]. The bifurcation model is characterized by a stenosis affecting all segments of the bifurcation: the PMB, DMB, and SB. A diameter stenosis of 60 % was chosen for the PMB and DMB, while the SB diameter stenosis varied among the different experiments in the clinical range (i.e. 40, 60, and 80 %) [23]. Consequently, these bifurcation stenoses represent 1,1,0 or 1,1,1 bifurcation lesions according to the Medina classification [24], which assigns a binary value (1, 0) to each of the three portions of the bifurcation (i.e. PMB, DMB, and SB) depending on whether they have more than (1) or less than (0) 50 % lesion. The lesions are eccentric with the plaque located in the inner arc of coronary vessels where low wall shear stress was present as a consequence of the vessel curvature (Fig. 1b). This modelling feature follows the findings by Iwami et al. [25]. The total lesion length was set to 12 mm for both branches, consistent with what was previously found in 1028 patients [26]. In order to take into account the curvature of the bifurcation due to the presence of the heart, the model was placed on a sphere with radius of 56.25 mm [27] which corresponds to a curvature ratio (i.e. vessel radius/radius of curvature) of 0.03. This value was in the range (0.02–0.50) as previously reported for the left coronary tree [28–30].

**Fig. 2:**
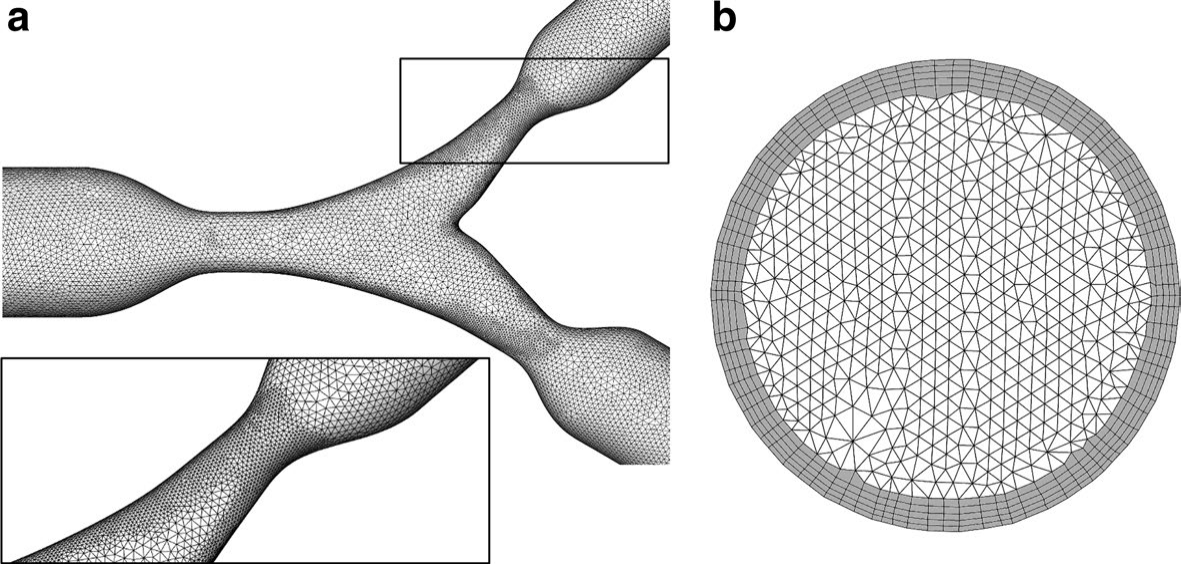
Details of the computational grid: **a** bifurcation region with smaller elements next to the stenosis; **b** inlet cross-section which is characterized by a prism layer (*dark grey colored*) close to the arterial lumen

An unstructured tetrahedral mesh was generated in ANSYS ICEM CFD v.15 (ANSYS Inc., Canonsburg, PA, USA) to discretize the bifurcation model. The fluid grid was characterized by smaller elements in the stenosis region and by a prism layer close to the arterial lumen to efficiently resolve the fluid dynamics quantities in the entire fluid domain (Fig. 2). The mesh element number was ~2,400,000 after a mesh independence study, which is briefly described at the end of this section.

### Fluid dynamic simulations

Since we aimed to calculate mean pressure values to derive the FFR, steady-state CFD simulations were performed, as done in previous studies [31, 32]. The finite volume software ANSYS Fluent v.15 (ANSYS Inc.) was used to carry out the fluid dynamics analyses. A hyperemic state was replicated by imposing a flow-rate of 120 mL/min at the inlet. This value is equal to three times the physiological value at rest (coronary flow reserve of 3) [33] that was obtained by solving the following equation [34]:

1 $$q = 1.43 \cdot d^{2.55}$$

where *q* is the flow and *d* is the diameter of the PMB (diameter of the inlet). Three different flow splits were applied at the bifurcation. First, a physiological (i.e. assuming the absence of stenoses) flow split was calculated following the relation between the diameter ratio of two daughter branches and the flow ratio through the bifurcation branches [34]:

2 $$\frac{{Q_{\text{SB}} }}{{Q_{\text{DMB}} }} = \left( {\frac{{d_{\text{SB}} }}{{d_{\text{DMB}} }}} \right)^{2.27}$$

where Q_SB_ and Q_DMB_ are the flow values and *d*_SB_ and *d*_DMB_ the diameters of the two daughter branches SB and DMB. The calculated flow split was 65 %:35 % for the DMB and SB, respectively. To account for population flow split variability, two additional flow splits were chosen with relative more (i.e. 55 %:45 %) and relative less (i.e. 75 %:25 %) flow through the SB outlet. The no-slip boundary condition was applied to the arterial wall, which was assumed to be rigid.

**Fig. 3:**
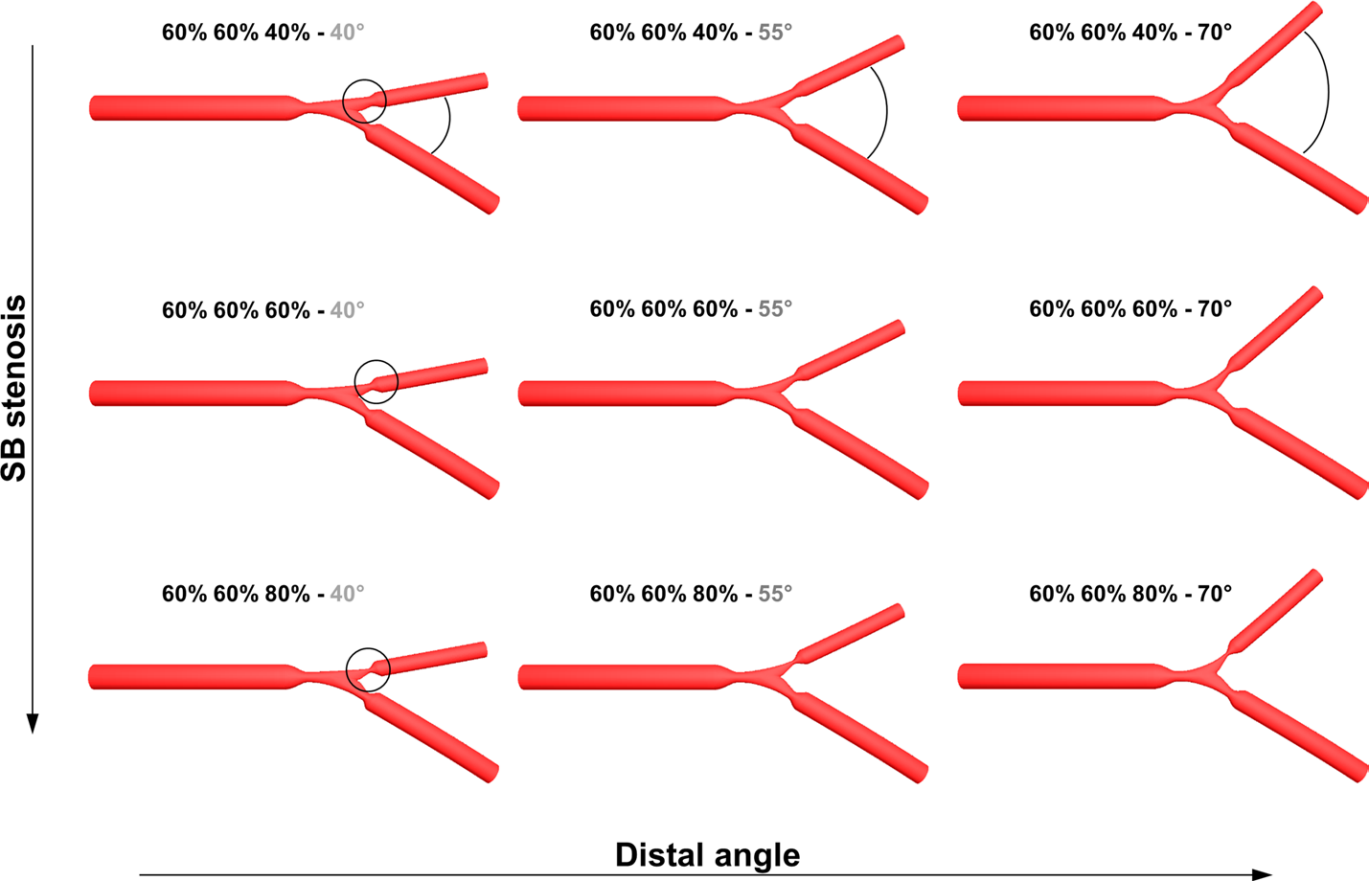
Investigated coronary bifurcation geometries. Each geometry is identified by the nomenclature “proximal main branch stenosis %, distal main branch stenosis %, side branch stenosis %—Distal angle (°)”

In summary, 27 simulations were performed by combining 3 distal angles (i.e. 40°, 55°, 70°), 3 degrees of SB stenosis (i.e. 40 %, 60 %, 80 %) while keeping PMB and DMB stenosis constant at 60 %, and 3 flow splits (55 %:45 %, 65 %:35 %, 75 %:25 % for the DMB and SB outlets, respectively) (Fig. 3).

The blood was modeled as a non-Newtonian fluid using the Carreau model:

3 $$\mu = \mu_{\infty } + \left( {\mu_{0} - \mu_{\infty } } \right) \cdot \left[ {1 + \left( {\lambda \cdot {\dot{\text{S}}}} \right)^{2} } \right]^{(n - 1)/2}$$

where *μ* is the dynamic viscosity, *μ*_*0*_ and *μ*_*∞*_ are the viscosity values as the shear rate goes to infinity and zero, respectively, ${\dot{\text{S}}}$ is the shear rate, *λ* is the time constant, and *n* is the Power-Law index. The following parameter values of the Carreau model were used [35]: *μ*_*∞*_ = 0.0035 Pa s, *μ*_*0*_ = 0.25 Pa s, *λ* = 25 s, and *n* = 0.25. A blood density of 1060 kg/m^3^ was chosen [35]. The flow was assumed to be laminar. Indeed, Reynolds number is ~235 at the inlet for all investigated cases. The bifurcation models with 80 % SB stenosis and flow split 55 %:45 % for the DMB and SB outlets, respectively, represent the extreme scenarios with Reynolds number of ~605 at the SB stenosis.

The solver settings and the computing platform specifications that were used to perform the CFD simulations are summarized in Table 1.

**Table 1:** Solver settings and computing platform specifications

Solver	
Type	ANSYS Fluent—pressure-based
Pressure–velocity coupling method	Coupled
Spatial discretization scheme—gradient	Least squares cell based
Spatial discretization scheme—pressure	Second order
Spatial discretization scheme—momentum	Second order upwind
Flow courant number	50
Explicit relaxation factors	
Momentum	0.3
Pressure	0.3
Residual value for convergence [35]	
Continuity	10^−5^
Velocity	10^−6^
Computing platform	1 node of a cluster (2 quad-core Intel Xeon E5620 @ 2.40 GHz, 24 GB RAM for each node, InfiniBand Mellanox for the main interconnections)
Number of computing cores	8

### Analysis of the results

Pressure in the PMB, more precisely at one diameter proximal to the stenosis, was set at the average aortic pressure for humans (100 mmHg) [32]. Pressure drops across the stenosis were calculated from PMB to DMB (ΔP_MB_ = 100 mmHg−pressure DMB) and from PMB to SB (ΔP_SB_ = 100 mmHg−pressure SB). Pressures in the DMB and SB were evaluated at cross-sections of one diameter distal to the stenosis, as indicated in Fig. 1. FFR of the DMB (FFR_MB_) was calculated as the ratio of the pressure in the DMB and the pressure in the PMB. FFR of the SB (FFR_SB_) was calculated as the ratio of the pressure in the SB and the pressure in the PMB.

The SB pressure drop was plotted against the absolute Q_SB_ for the 9 simulations in which the SB stenosis is 80 %. The relationship between ΔP_SB_ and Q_SB_ was described for the cases with SB stenosis of 80 % as ΔP = A*Q* + B*Q*^2^. The first term (A) of these relationship describes the viscous friction losses over the stenosis according to Pouseuille’s law while the second term (B) describes the pressure losses caused by convective acceleration along the narrowing according to Bernoulli’s law [36]. The goodness of the quadratic fits was evaluated by calculating the root mean squared error (RMSE), which is defined as:

4 $$RMSE = \sqrt {\frac{1}{n - m}\mathop \sum \limits_{i = 1}^{n} \left( {y_{i} - \hat{y}_{i} } \right)^{2} }$$

where *n* is number of response values, *m* the number of fitted coefficients estimated from the response values, *y*_*i*_ is the ith value of the variable to be predicted, and $\hat{y}_{i}$ is the predicted value of *y*_*i*_. Smaller values of RMSE indicate that the observations are closer to the fitted line.

In order to visualize the flow patterns inside the coronary bifurcations, the local normalized helicity was calculated. This quantity has been widely adopted in the cardiovascular field of biomechanical engineering to describe the arrangement of fluid streams into spiral patterns [35, 37–42]. Positive and negative local normalized helicity values point out clockwise and counter-clockwise rotating fluid structures along the main flow direction, respectively. Additionally, to quantify the strength of the spiral flow structures that develop in the bifurcation, the helicity intensity was computed, as previously done in several recent numerical studies on coronary arteries, carotid bifurcations, and aortas [37, 42, 43].

### Mesh independence study

To ensure the independence of the results from the mesh size, a mesh independence study was conducted on one representative geometry (i.e. case with stenosis degree of 60 % in the SB, distal angle of 70°, and flow split 55 %:45 % for the DMB and SB outlets, respectively). Three meshes were created, from a coarser to a finer one, by increasing the element number by a factor ~1.5 between each consecutive mesh: 1,671,949, 2,390,756, and 3,671,302 elements. The meshes were compared by evaluating the maximum velocity in the fluid domain and the pressure drops across the stenosis from PMB to DMB (ΔP_MB_) and from PMB to SB (ΔP_SB_). Results are reported in Table 2. Since the percentage difference between the intermediate and the finest mesh was lower than 0.5 % for the maximum velocity and 0.15 % for the pressure drops, the intermediate mesh (~2,400,000) was considered sufficiently accurate for the calculations.

**Table 2:** Grids and results of the mesh independence study

Number of elements	Max velocity		ΔP_MB_		ΔP_SB_	
	[m/s]	Perc. diff (%)	[mmHg]	Perc. diff (%)	[mmHg]	Perc. diff (%)
1,671,949	2.10	1.05	12.07	0.55	18.54	0.17
2,390,756	2.12	0.36	12.12	0.13	18.58	0.03
3,671,302	2.13	–	12.13	–	18.57	–

The percentage difference is calculated with respect to the finest mesh

## Results

**Fig. 4:**
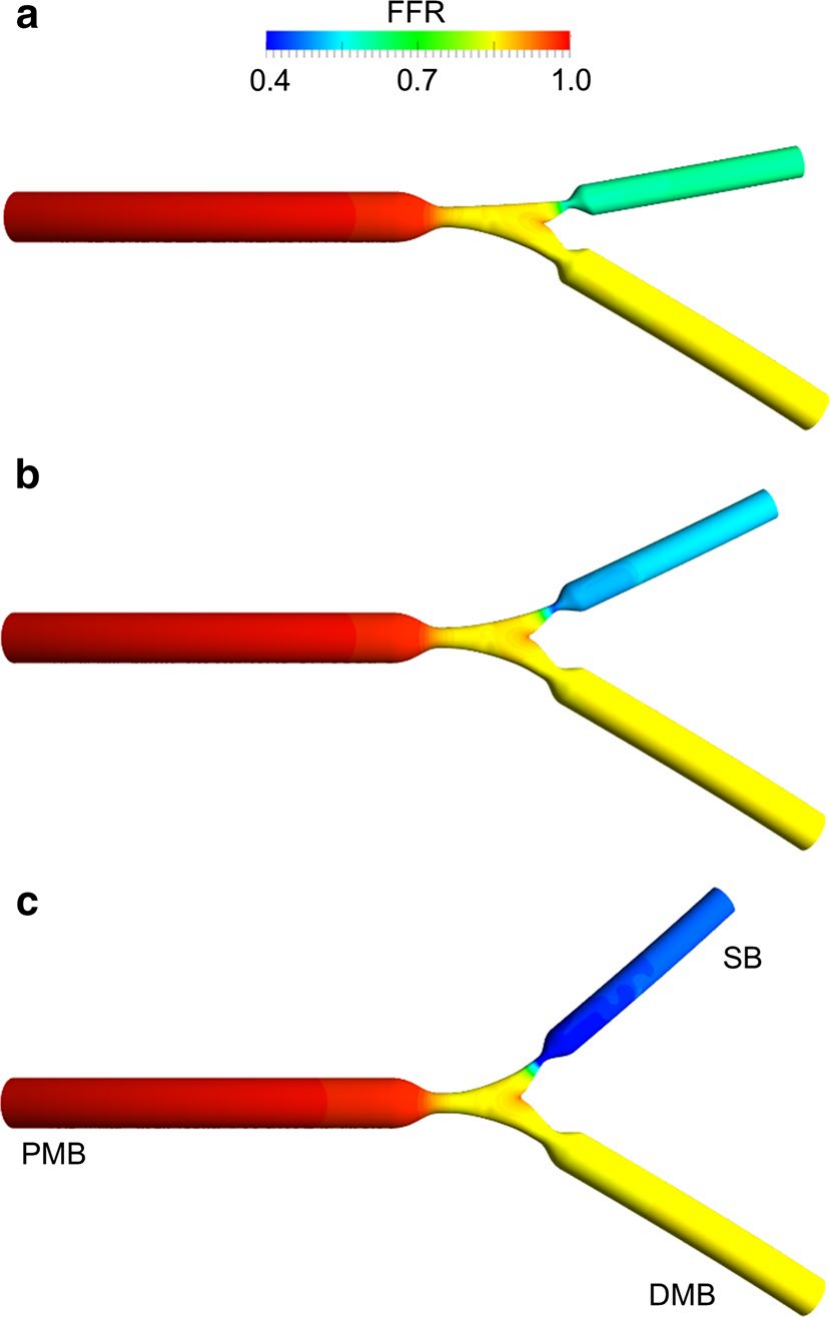
Contour plots of FFR for cases with 80 % side branch stenosis and flow split of 65 %:35 % (for the distal main branch and side branch outlets, respectively), which corresponds to 42 mL/min side branch flow-rate. The distal angle was variable: 40° (**a**), 55° (**b**), 70° (**c**). The location of the proximal main branch (PMB), distal main branch (DMB), and side branch (SB) is indicated in **c**. Note that with increasing distal bifurcation angle, FFR in the side branch decreases

Table 3 shows the pressure drops and calculated FFR values for the DMB and SB for the 27 simulations we have performed. In the presence of mild SB stenosis of 40 %, the bifurcation angles have only limited influence on the SB pressure drop. Irrespective of the assumed flow split, there is a difference in FFR_SB_ of only 0.02 when comparing the largest and smallest bifurcation angles. When there is an intermediate SB stenosis of 60 %, the bifurcation angle does have some influence on the FFR_SB_. The differences in FFR_SB_ between the largest and smallest bifurcation angles are 0.03 (flow split of 75 %:25 %), 0.04 (flow split of 65 %:35 %) and 0.05 (flow split of 55 %:45 %), respectively. However, when the SB stenosis is more severe (80 %), the FFR_SB_ is influenced significantly by the bifurcation angle. The differences between the largest and smallest SB angles in FFR_SB_ were 0.13 (flow split of 75 %:25 %), 0.22 (flow split of 65 %:35 %, see Fig. 4) and 0.33 (flow split of 55 %:45 %), respectively.

**Table 3:** Pressure drop across the stenosis from proximal to distal main branch (ΔP_MB_), fractional flow reserve in the main branch (FFR_MB_), pressure drop across the stenosis from proximal main branch to side branch (ΔP_SB_), and fractional flow reserve in the side branch (FFR_SB_) for all investigated cases

Case (SB %DS—angle α—%SB flow)	ΔP_MB_ (mmHg)	FFR_MB_	ΔP_SB_ (mmHg)	FFR_SB_
40 %—40°—25 %	14.89	0.851	7.10	0.929
40 %—55°—25 %	14.93	0.851	8.37	0.916
40 %—70°—25 %	14.71	0.853	9.26	0.907
40 %—40°—35 %	13.07	0.869	7.36	0.926
40 %—55°—35 %	12.82	0.872	8.69	0.913
40 %—70°—35 %	12.58	0.874	9.54	0.905
40 %—40°—45 %	11.55	0.885	7.80	0.922
40 %—55°—45 %	11.12	0.889	9.19	0.908
40 %—70°—45 %	10.93	0.891	10.04	0.900
60 %—40°—25 %	14.85	0.851	9.35	0.906
60 %—55°—25 %	15.20	0.848	10.99	0.890
60 %—70°—25 %	15.41	0.846	12.80	0.872
60 %—40°—35 %	13.26	0.867	11.09	0.889
60 %—55°—35 %	13.31	0.867	13.34	0.867
60 %—70°—35 %	13.51	0.865	15.18	0.848
60 %—40°—45 %	12.06	0.881	13.81	0.862
60 %—55°—45 %	11.89	0.881	16.73	0.833
60 %—70°—45 %	12.12	0.879	18.58	0.814
80 %—40°—25 %	15.20	0.848	22.53	0.775
80 %—55°—25 %	15.27	0.847	30.54	0.695
80 %—70°—25 %	15.63	0.844	36.03	0.640
80 %—40°—35 %	13.76	0.862	35.50	0.645
80 %—55°—35 %	13.55	0.864	49.61	0.504
80 %—70°—35 %	13.87	0.861	57.72	0.423
80 %—40°—45 %	12.69	0.873	52.66	0.473
80 %—55°—45 %	12.30	0.877	74.16	0.258
80 %—70°—45 %	12.60	0.874	85.98	0.140

*SB* side branch; *%DS* percentage diameter stenosis; *angle α* distal bifurcation angle; *%SB flow-rate* percentage of inlet flow-rate through side branch

**Fig. 5:**
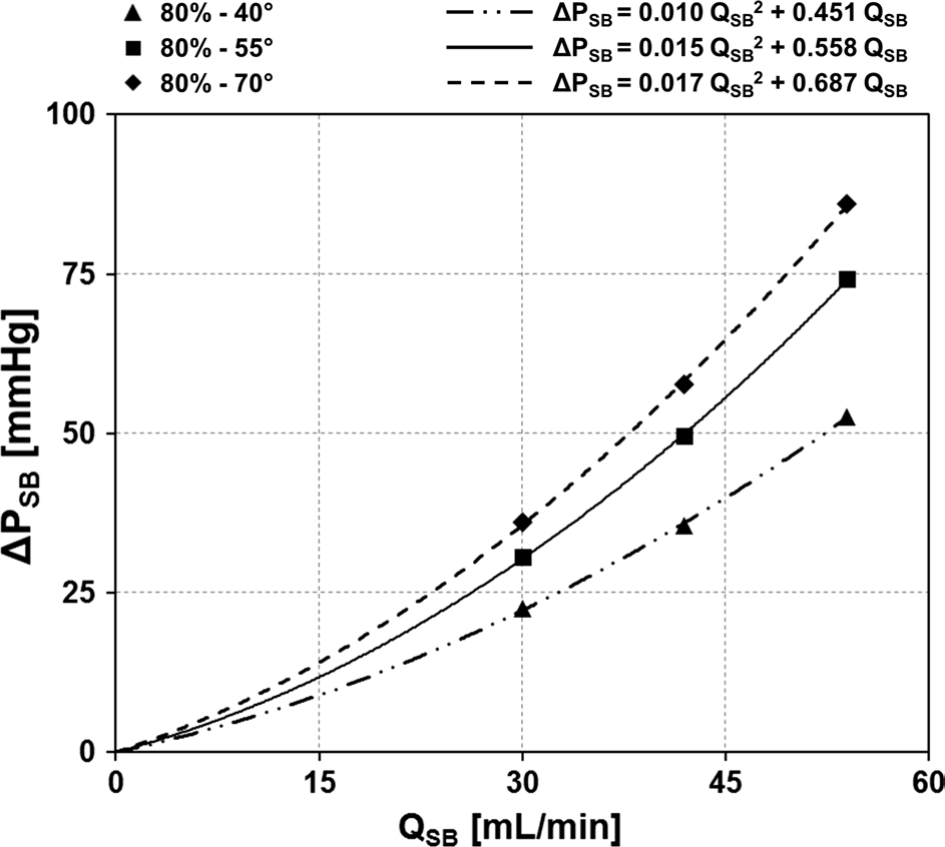
Pressure drop across the stenosis from proximal main branch to side branch (ΔP_SB_) against side branch flow-rate (Q_SB_) for all cases with 80 % side branch stenosis. The pressure drop of each bifurcation case are interpolated using a quadratic polynomial *curve* reported above the *plot*

Figure 5 shows the ΔP_SB_ plotted against the absolute Q_SB_ for the 9 simulations in which the SB stenosis is 80 %. A good quadratic fit was obtained for cases with different distal angle, as highlighted by the small values of RMSE (0.58, 0.47, and 0.73 mmHg for cases with 40°, 55°, and 70° distal angle, respectively). Both terms in the equation defining the ΔP_SB_−Q_SB_ relationship (i.e. ΔP = A *Q* + B *Q*^2^) were larger with increasing bifurcation angles, resulting in steeper curves describing the ΔP_SB_ and Q_SB_ relationship, suggesting that the stenosis resistance of 80 % SB stenosis increases in larger bifurcation angles.

**Fig. 6:**
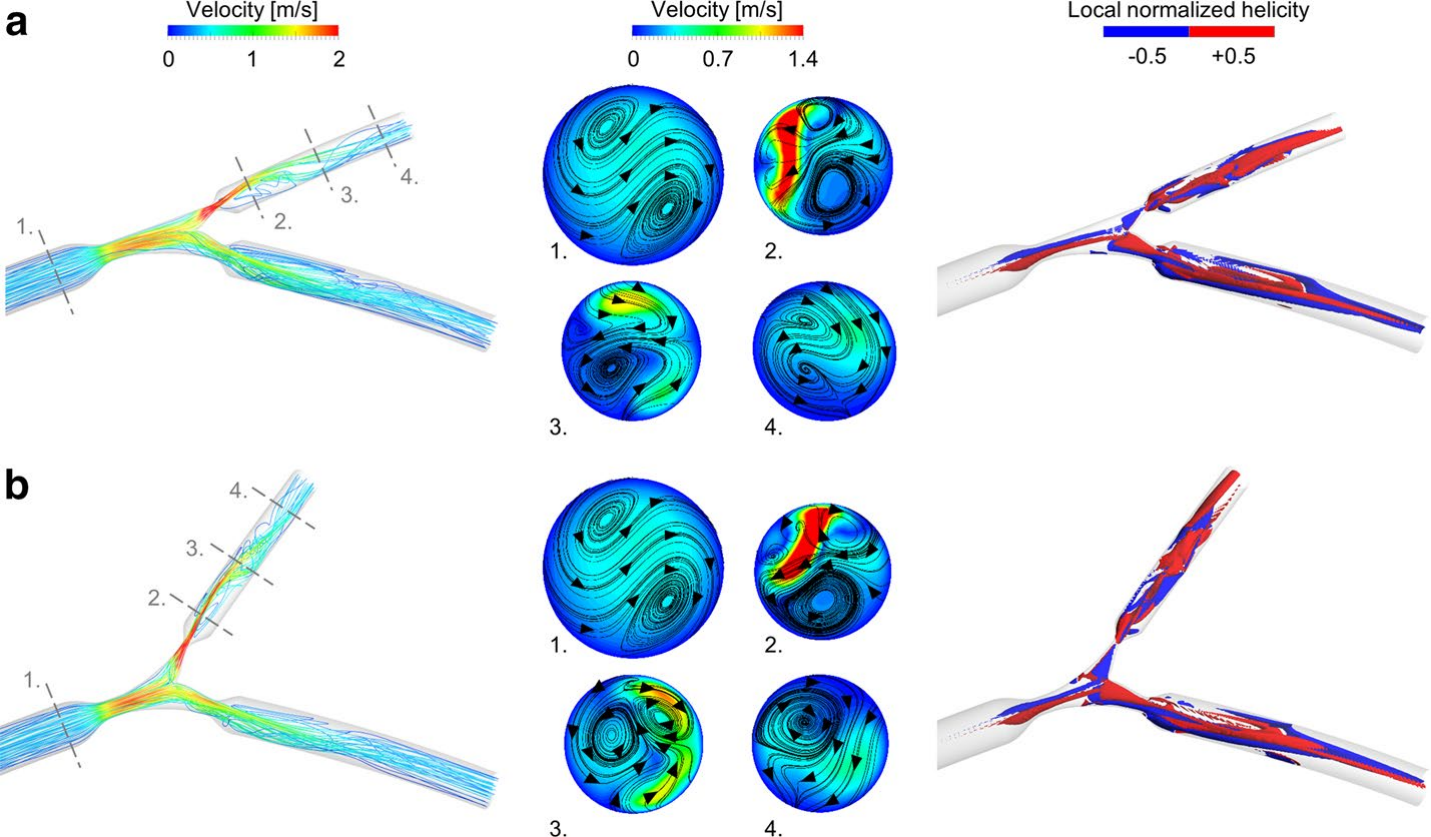
Velocity pathlines (*left*), velocity contours with in-plane velocity vectors at selected cross sections (*center*), and isosurfaces of local normalized helicity (*right*) for cases with 80 % side branch stenosis, flow split of 65 %:35 %, and distal angle of 40° (**a**) and 70° (**b**). Positive and negative values of local normalized helicity indicate counter-rotating flow structures

**Fig. 7:**
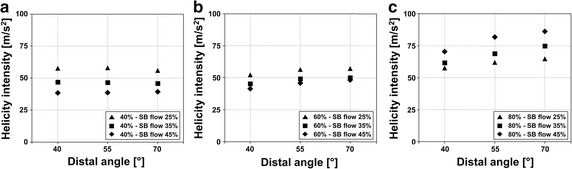
Helicity intensity against distal angle for all cases with side branch stenosis of 40 % (**a**), 60 % (**b**), and 80 % (**c**). The *symbols* indicate cases with different flow split

Figure 6 shows the complex flow patterns in the bifurcation. In particular, a jet with high velocity is visible in the SB, downstream of the stenosis (Fig. 6a). Recirculations can also be observed in the same region. The vessel curvature generates secondary flows in all segments, with more complex patterns in the SB, as highlighted by the in-plane velocity pathlines at SB selected cross-sections (Fig. 6b). Complex spiral flow patterns with clockwise and counterclockwise rotating fluid structures originate in the stenosed bifurcation region and develop into the two daughter vessels (with 60 % PMB and DMB stenosis and 80 % SB stenosis, see Fig. 6c). These spiral flow patterns were observed in all cases and they were more pronounced in the models with large bifurcation angle and severe SB stenosis, as shown by Fig. 7. In this figure, the helicity intensity versus the distal angle is reported for all cases with different SB stenosis. Helicity intensity is not affected by distal angle when SB stenosis is 40 % (Fig. 7a). On the contrary, helicity intensity increases when distal angle becomes larger for cases with 60 and 80 % SB stenosis, for all flow splits applied at the bifurcation outlets (Fig. 7b, c).

**Fig. 8:**
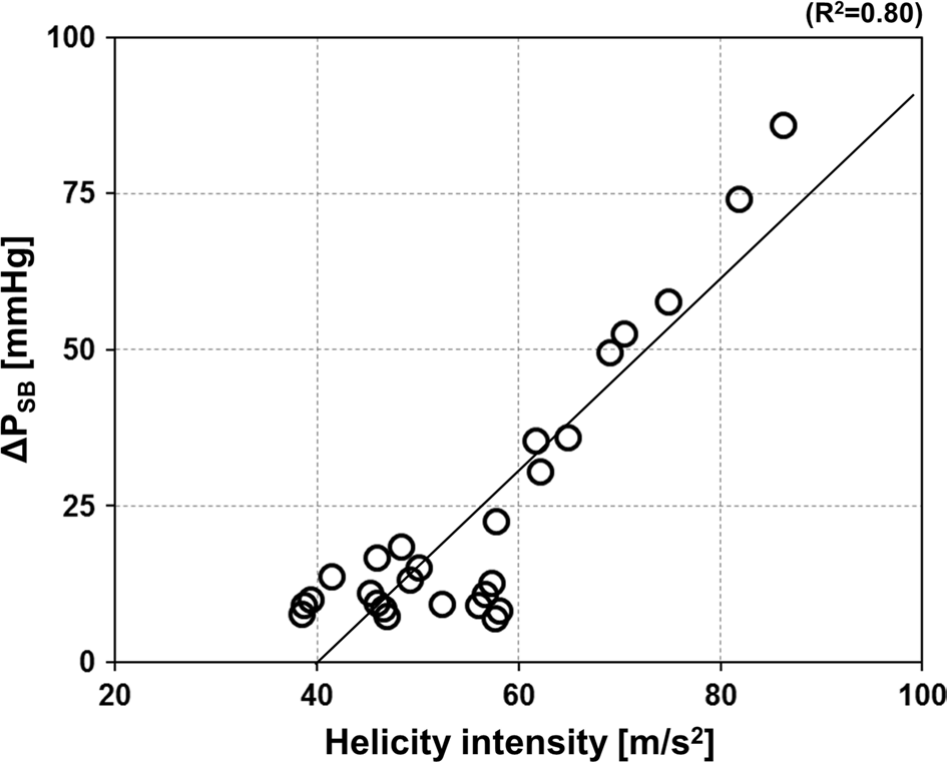
Scatter plot showing the pressure drop across the stenosis from proximal main branch to side branch (ΔP_SB_) against helicity intensity calculated for each case. The line shows the linear regression fit. R^2^ is the determination coefficient

Finally, to investigate the relation between the SB pressure drop and the complex flow patterns that characterize the stenosed bifurcation models, the SB pressure drop of each investigated case was plotted against the corresponding helicity intensity value (Fig. 8). A good linear correlation (R^2^ = 0.80) between the two quantities was found.

## Discussion

In the present study, we evaluated the influence of bifurcation angle and SB stenosis on pressure drop and corresponding FFR. The main finding of the study is that in models of bifurcation lesions with 60 % MB stenosis and 80 % SB stenosis, the SB pressure drop is higher suggesting higher flow resistance (i.e. curves describing the ΔP_SB_−Q_SB_ relationship being steeper) when the distal bifurcation angle is larger. However, when the SB stenosis is mild (40 %), the SB resistance is minimally influenced by the bifurcation angle.

In interventional cardiology, FFR has become a feasible invasive measurement to assess potential myocardial ischemia under high work load by calculating the ratio between the pressure distal to the coronary artery stenosis and the aortic pressure under hyperemic conditions. Despite the widespread acceptance of FFR, a deeper comprehension of its physiological basis and diagnostic features is needed to better understand the meaning of the FFR values measured in each patient, in particular when bifurcation lesions are treated [36]. CFD simulations can provide useful information by systematically calculating pressure drops and FFR values in coronary bifurcation models under different scenarios. In this study we evaluated the influence of bifurcation angle and SB stenosis on pressure drops and corresponding FFR. By plotting the ΔP_SB_ values against the absolute Q_SB_ values (Fig. 5), we could evaluate the ΔP_SB_−Q_SB_ relationships. We showed that the curves describing these ΔP_SB_−Q_SB_ relationships become steeper in larger bifurcation angles when SB stenosis is severe. As a consequence, we also found that FFR_SB_ is significantly influenced by the bifurcation angle in case of severe SB stenosis.

Our CFD analyses highlighted also the complex interplay between hemodynamics and vessel geometry. Indeed, the geometric features of coronary bifurcations dictate the local hemodynamic environment, which influences the process of atherosclerotic plaque initiation and progression [44]. While previous numerical studies on coronary bifurcations focused on the relation between geometric features such as vessel tortuosity and bifurcation angle with wall shear stress descriptors [12, 15, 44, 45], in this work we investigated the impact of bifurcation angle and degree of stenosis on pressure drop (and FFR) and on the bulk flow, helicity under hyperemic conditions. The results of our study revealed complex hemodynamics in all investigated bifurcations with marked secondary flows and recirculation areas in the SB. Counter-rotating helical flow structures were evident in the bifurcation region and in the branches (Fig. 6). These hemodynamic patterns were caused by the combined effect of the curvature of the bifurcation, the presence of the stenosis, and also the bifurcation angle. In particular, larger bifurcation angles resulted in more pronounced helical flow structures (i.e. higher helicity intensity, Fig. 7) when SB stenoses of 60 or 80 % were present. Additionally, a good correlation between the pressure drop in the SB and helicity intensity was found (Fig. 8), suggesting that marked helical flow structures caused by the specific geometric features of the vessel result in higher pressure drops, reflecting higher resistance. This result is in agreement with the pressure-flow relationships (Fig. 5) and the previous CFD findings obtained for patient-specific coronary segments under resting conditions [42].

Currently, FFR measurements are applied to examine the functional severity of a stenosis in order to decide for PCI with promising results compared to the classical angiography [1]. Furthermore, FFR measurements are also used to judge treatment result. During treatment of a bifurcation lesion by stenting of the MB, SB compromise is often observed when the angle between the MB and the SB is small. Interestingly, a poor correlation between ostial SB narrowing due to PCI of the MB and FFR measurements, was observed [46]. These findings together with the findings of the current study suggest that the bifurcation angle also plays an important role to predict SB flow compromise after MB stenting due to higher SB flow resistances when the bifurcation angle is larger. However, this remains speculative and future flow simulation studies are needed to investigate the influence of the bifurcation angle on the hemodynamic impact of the SB after MB stenting. The complex interplay between the bifurcation angle, the degree of stenosis, and the hemodynamics can render pressure drop unreliable for examination of SB perfusion.

In this study, idealized, population-based bifurcation models were used. Although the geometric dimensions, including the curvature of the heart, were taken from the literature, coronary flow may behave differently in true human coronary anatomy. Currently, it is possible to perform CFD simulations on three-dimensional patient-specific human coronary anatomies reconstructed from computed tomography or quantitative coronary angiography and/or intravascular imaging [35, 42, 47–49]. Although local hemodynamics (e.g. secondary flows and wall shear stress) cannot be measured in vivo in coronary arteries, pressure and flow (velocity) measurements can be done and used as boundary conditions for the CFD models. However, the advantage of the use of population-based over patient-specific models is that it is possible to vary one specific anatomic component, such as the bifurcation angle, while keeping other variables constant. Consequently, the direct influence of that specific anatomic component on the local hemodynamics can be investigated. Additionally, as demonstrated in a previous numerical study [11], hemodynamic results in idealized bifurcation geometries are consistent in location and magnitude with those of the patient-specific anatomies that the idealized models represent.

In daily clinical practice, FFR values are in general not obtained in bifurcations in which the PMB is involved since interpretation of the FFR value will be hampered by the impossibility to distinguish relative contribution of the proximal and distal stenosis to the pressure drop. However, by displaying the curves describing the ΔP_SB_−Q_SB_ relationships of the 80 % SB stenosis for each bifurcation angle separately, we were able to describe the SB stenosis resistances, which were clearly influenced by SB angle when SB stenosis was 80 %. It is likely that such resistances play a role in the flow distribution to the SB and DMB and thus play a role in SB compromise, although future studies are needed to further investigate this.

Moreover, we imposed flow under maximal hyperemia as inlet boundary condition. Hereby we assumed the microvasculature being healthy with the distal resistances (i.e. microvascular resistances) being negligibly low. However, impaired microvascular function would impact the microvascular resistance resulting in a different assumed hyperemic flow and thus would have an impact on the calculated FFR values. Furthermore, the stenosis at the bifurcation would also result in a reduced flow, especially when the stenosis becomes more severe. Therefore, the absolute FFR values we have obtained under hyperemic conditions are higher than what would be expected in vivo. However, by showing the ΔP_SB_−Q_SB_ relationships using the imposed flow assumptions, we were still able to draw conclusions on the influence of bifurcation angle on the SB stenosis resistance.

We assumed three different flow splits, one representing the natural flow split in case there would have been no stenosis, one with relative more flow diverted to the SB, and one with relative less flow diverted towards to SB. Although physiological (i.e. in case of no stenosis) flow split ratios at coronary bifurcations are well described in the literature [34, 50], less is known about the flow split under pathological circumstances (i.e. with different stenosis degrees). In the reality, the flow split depends on the distal resistances, which are related to the patient-specific condition of the myocardium. Future studies using lumped parameter models (LPM), quantifying the entire coronary circulation (including the microvasculature) based on a hydraulic-electrical analogue, can be used to estimate the flow split under different circumstances. Such model could also take into account the influence of collateral flow. The specific ΔP_SB_−Q_SB_ relationships found under the different circumstances (SB and DMB diameter stenosis, bifurcation angles, etc.) can be included in such LPM models to automatically calculate the flow splits [51]. Furthermore, the investigation of the coronary branch steal phenomenon [52] and its influence on FFR_SB_ values by using these LPM models would be of particular interest.

We assumed laminar flow conditions for all our calculations. However, in the most extreme scenarios (i.e. bifurcation models with 80 % SB stenosis and flow split 55 %:45 % for the DMB and SB outlets, respectively) Reynolds number was ~605 at the SB stenosis. This value is at the borderline in the range between 500 and 1000, for which flow instabilities were observed in non-realistic axisymmetric stenosed vessels [53, 54]. In a more realistic geometry, namely a carotid bifurcation, transitional flow was only observed close to the stenosis for higher local Reynolds numbers (peak Reynolds number of ~1200 at the stenosis) [55]. Thus, in our study the flow was assumed to be laminar in all cases for comparative purposes and to simplify numerics, as previously done in [13]. This assumption is conservative because it might result in a slightly underestimation of the pressure drops for the extreme cases, leading to the calculation of slightly higher FFR values.

Finally, it should be highlighted that the models are rigid and fixed. Although these limitations might have effects on near-wall hemodynamics quantities like wall shear stress, the pressure values are minimally affected by the wall movement, as shown in a recent study [56].

## Conclusions

In the present study, we evaluated the influence of bifurcation angle and SB stenosis on pressure drops and corresponding FFR. Our CFD simulations showed that, in bifurcation lesions with 60 % MB stenosis and 80 % SB stenosis, the SB pressure drop increases implying that the flow resistance increases when the distal bifurcation angle is larger. When the SB stenosis is mild (40 %), the SB resistance is only minimally influenced by the bifurcation angle, with differences which are not clinically meaningful. Our findings also highlighted the complex interplay between anatomy, pressure drops, and blood flow helicity in bifurcations. Future studies should focus on how the anatomic specific SB resistances will influence the flow split to the DMB and SB, respectively.

## Abbreviations

PCI: percutaneous coronary intervention
FFR: fractional flow reserve
SB: side branch
MB: main branch
CFD: computational fluid dynamics
LAD: left anterior descending coronary artery
PMB: proximal main branch
DMB: distal main branch
RMSE: root mean squared error
LPM: lumped parameter model
